# Hydrogen Sulfide Gas Detection via Multivariate Optical Computing

**DOI:** 10.3390/s18072006

**Published:** 2018-06-22

**Authors:** Bin Dai, Christopher Michael Jones, Megan Pearl, Mickey Pelletier, Mickey Myrick

**Affiliations:** 1 Sensor Physics Department, Halliburton Company, 3000 N. Sam Houston Pkwy E., Houston, TX 77032, USA; Bin.Dai2@halliburton.com (B.D.); Megan.Pearl@halliburton.com (M.P.); Mickey.Pelletier@Halliburton.com (M.P.); 2 Department of Chemistry and Biochemistry, University of South Carolina, Columbia, SC 29208, USA; myrick@sc.edu

**Keywords:** H_2_S, multivariate optical computing, multivariate optical element, downhole optical sensor, UV spectroscopy

## Abstract

Hydrogen-sulfide gas is a toxic, colorless gas with a pungent odor that occurs naturally as a decomposition by-product. It is critical to monitor the concentration of hydrogen sulfide. Multivariate optical computing (MOC) is a method that can monitor analytes while minimizing responses to interferences. MOC is a technique by which an analogue calculation is performed entirely in the optical domain, which simplifies instrument design, prevents the drift of a calibration, and increases the strength and durability of spectroscopic instrumentation against physical perturbation when used for chemical detection and identification. This paper discusses the detection of hydrogen-sulfide gas in the ultraviolet (UV) spectral region in the presence of interfering gaseous species. A laboratory spectroscopic measurement system was set up to acquire the UV spectra of H_2_S and interference gas mixtures in high-pressure/high-temperature (HPHT) conditions. These spectra were used to guide the design and fabrication of a multivariate optical element (MOE), which has an expected measurement relative accuracy of 3.3% for H_2_S, with a concentration in the range of 0–150 nmol/mL. An MOC validation system with the MOE was used to test three samples of H_2_S and mercaptans mixtures under various pressures, and the relative accuracy of H_2_S measurement was determined to be 8.05%.

## 1. Introduction

Hydrogen sulfide (H_2_S) gas is a corrosive and potentially life-threatening gas. The detection and handling of H_2_S has become a growing challenge in the petroleum industry [1,2]. In upstream operations—including oilfield exploration, development, and production—it is critical to detect and monitor the level of H_2_S. H_2_S can cause corrosion and cracking of metals, so the production of fluid with H_2_S may require costly special production equipment. Special materials and equipment are required to produce petroleum with H_2_S, thereby substantially increasing the cost of petroleum production compared to reservoirs without H_2_S [3,4,5]. The decision to produce an oil and gas reservoir is, in part, dependent on production difficulties that may occur, including those dictated by the presence of H_2_S. To determine the concentration of H_2_S within a reservoir, the downhole fluid sample is often acquired from the reservoir and transported to the surface. The sample is then sent to a laboratory, where conventional analytical technologies, such as gas chromatography and lead acetate H_2_S sensing tape method, are used to measure the concentration of H_2_S. Unfortunately, owing to its corrosive nature, H_2_S often reacts with the equipment used to acquire the sample and the sample bottle itself. As such, laboratory measurements tend to significantly underestimate the true H_2_S level of the formation fluid [1,2]. The presence or actual concentration of H_2_S is not discovered until the production stage after a large capital investment has been spent. Because retrofitting production is extremely expensive, and sometimes not possible, a field development program usually assumes the worst-case scenarios if any H_2_S is detected in the downhole sample bottle. The cost of over-engineering could be mitigated if an in situ method for H_2_S measurement were available; however, there is currently no method for H_2_S measurement within the HPHT environment of subterranean petroleum wells. Therefore, a method for the real-time, accurate downhole detection of H_2_S is much desired.

Petroleum gas consists of both hydrocarbon and non-hydrocarbon components. Hydrocarbon gas components are usually methane, ethane, propane, normal and iso-butane, and normal and iso-pentane [6,7]. Trace liquid components of higher molecular weight than the pentanes, called the C6+ fraction, exist only in trace quantities of less than 1 mol %. The gaseous C6+ fraction contains saturated hydrocarbons and aromatic benzene, ethylbenzene, toluene, and xylene hydrocarbons [8]. The hydrocarbon concentration decreases exponentially as a function hydrocarbon molecular weight [7,9]. The inorganic gas composition concentration usually consists of carbon dioxide, water, nitrogen, and mercaptans [6,8].

In the last two decades, many different sensor types for measuring H_2_S have been developed and commercialized. Sensors based on optical absorption spectroscopy include tunable diode laser absorbance spectroscopy (TDLAS) [10] or FTIR method [11]. Sensors based on metal oxide semiconductors are also widely used for H_2_S monitoring [12,13]. Cavity-enhanced Raman spectroscopy methods have also been explored for gas phase H_2_S measurement [14]. Although these sensors are useful for many applications, none of them can be used for real time H_2_S measurement in downhole harsh condition due to either the reliability issues of sensors at high temperature and pressure or large size of sensors that will not fit the tool used for downhole measurement application.

The method using UV spectroscopy for hydrogen sulfide detection has been explored [15]. The strong feature from 167 to 250 nm for H_2_S is very close to the experimental band maximum. The transition is assigned as a strong Rydberg 2b_1_-->4s overlapping with a much more valence-like 2b_1_-->3d_xz_ system [16]. UV spectroscopy been proposed as a method for H_2_S gas detection in petroleum gas samples within gas reservoirs owing to the strong signal in the spectral range from 190 to 240 nm and small interference from petroleum gas components [17,18]. Mercaptans are the only hydrocarbon gases present providing interference at normal component concentrations [19,20]. H_2_S has been measured at high temperatures [21,22] and pressures [17]. However, pressure measurements of 15,000 psi have not been attained, which are typical of petroleum reservoirs.

Multivariate optical computing (MOC) is an optical analysis technique whereby spectral information is computed in the optical domain, thereby providing quantitative chemical or physio chemical information [23]. At the heart of the system is the multivariate optical element (MOE) for which the transmission pattern is a dot-product regression vector. As light traverses the MOE and strikes a detector, an analog dot product of the regression vector is computed with respect to the spectral pattern. MOC has been shown to provide similar analysis accuracy as the laboratory spectrometer from which an MOE is designed [24,25,26,27]. Proof-of-concept systems have been developed for visible, near-infrared, and mid-infrared systems [28,29]. The technique has been shown to work for Raman applications, florescent applications [23,30,31,32,33,34,35], absorbance applications [24,29,36,37], reflectance applications [28], and hyperspectral imaging [30,38].

## 2. Theory of MOC

**Figure 1:**
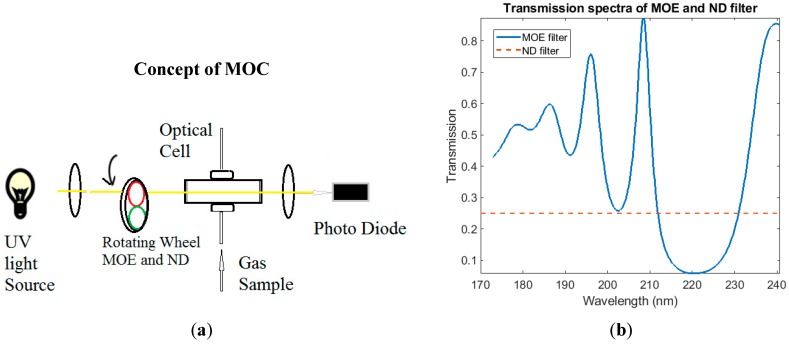
(**a**) The schematic concept of MOC sensor; (**b**) example transmission spectra of MOE and ND filter.

Figure 1 illustrates the MOC concept for sample analysis. A typical MOC system includes a broad spectrum light source, sample cells, multivariate optical element (MOE), neutral density filter (ND filter), and optical detectors. The integrated detector signal (d) through each channel (either MOE or ND filter) is the convolution and integration of the spectra of the sensor components, which includes lamp emission spectrum (*L*), sample transmission spectrum (s), filter transmission spectrum (*T_MOE_* or *T_ND_*), and a detector spectral sensitivity profile (*R*), as expressed in Equations (1) and (2).

(1) $$d_{A}=\int_{\lambda 1}^{\lambda 2}T_{MOE}(\lambda )L(\lambda )s(\lambda )R(\lambda )d\lambda$$

(2) $$d_{B}=\int_{\lambda 1}^{\lambda 2}T_{ND}(\lambda )L(\lambda )s(\lambda )R(\lambda )d\lambda =b\int_{\lambda 1}^{\lambda 2}L(\lambda )s(\lambda )R(\lambda )d\lambda$$

where: *b* is transmission rate of flat ND filter ( 25% transmittance, etc.), *λ*1 and *λ*2 are the starting and ending wavelength of spectral region of interest, respectively.

To design the MOE filter, a sufficient number of transmission spectra of representative samples must be collected as training set using a conventional spectrometer (dispersive, grating, or Fourier transform). Assuming that spectra of *m* samples are recorded and each spectrum consists of *n* data points (depends on the resolution of the spectrum and spectral range), a training transmission spectra data matrix (*S*) with a size of *m × n* can be obtained. To simplify the calculation, the training spectra data matrix is convolved with a light-source spectra profile (*L*) and detector responsivity profile (*R*) to obtain convolved sample transmission spectra ($\bar{S}$)

(3) $$\bar{S}(\lambda )=S(\lambda )L(\lambda )R(\lambda )$$

As has been described, the goal of sensor design is to create one MOE with unique spectral transmittance patterns (*T*) such that the linear combination of the detector signals through MOE and the ND filter can best estimate the property of the sample (such as chemical composition) [24]. The estimation of the property of interest can be obtained through linear regression of the ratio of detector signals to the known property of interest in the training set.

(4) $$\hat{y}=\beta \frac{d_{a}}{d_{b}}+\alpha =\beta \frac{\bar{S}•T_{MOE}}{b\sum \bar{S}}+\alpha =\beta (\frac{1}{b}\times \frac{\bar{S}}{\sum \bar{S}}•T_{MOE})+\alpha =\beta (\hat{S}•T_{MOE})+\alpha$$

where,

- $\hat{y}$ is the estimated value of the property of interest
- $\hat{S}$ is the normalized convolved spectra of samples
- *d_a_* and *d_b_* are the detector responses of MOE channel and ND channel, respectively.
- α is the calibration offset and β is the weight coefficient
- ‘•’ is the dot product operator

Two primary considerations must be made when designing an MOE. First, the designed MOE must minimize the difference between true values and predicted values of the property of interest (minimizing the mean squares error, optimizing the measurement accuracy). Second, the designed MOEs must minimize the prediction uncertainty (minimizing the propagation of detector noise to concentration prediction, improving the measurement precision). Because the detector noise characterization is known for a given sensor, the minimization aims to reduce the weight coefficient (β) to minimize noise propagation.

Based on the previously described considerations, the design optimization algorithm can be formulated as a constrained least-squares regression (ridge regression), as shown in Equation (5).

(5) $$T_{opt}=\arg\min({\left\|y-\hat{y}\right\|}_{2}+\theta \beta^{2})$$

(6) $$\hat{y}=\beta (\hat{S}•T_{MOE})+\alpha$$

where,

- *y* is known value of the property of interest in the training set
- ${\left\|\;\right\|}_{2}$ is the L_2_ norm
- *T*_opt_ is the optimal MOE transmission profile
- *T*_MOE_ is the MOE transmission spectrum

The *θ* is the penalty term that is predetermined based on the MOC sensor’s signal to noise characteristics. It is inversely proportional to the detector’s signal-to-noise ratio (S/N); for sensors with lower S/N, the *θ* term will be larger to increase the penalty on the regression weighting (*β*).

**Figure 2:**
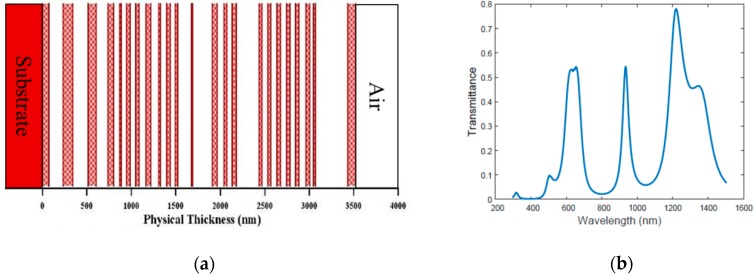
(**a**)Thin-film structure of an example MOE; (**b**) corresponding transmission spectrum of the MOE.

The transmittance pattern of MOE is determined by its thin-film stack structure. By alternating two materials with different indices of refraction and absorption coefficients and varying the thickness of each layer, a thin-film filter with different transmittance patterns can be generated. Essentially, the optimization objective provided in Equation (5) is achieved by optimizing the number of layers and the thicknesses of each layer. Figure 2 shows an example MOE‘s thin-film stack structure and its transmittance pattern. Once the design of the thin-film stack structure is completed, an MOE filter is fabricated layer by layer using a vapor-deposition method, such as ion-assisted E-beam deposition, physical vapor deposition, sputtering, or chemical vapor deposition [26,39].

The fabricated MOE can be installed into the MOC sensor system demonstrated in Figure 1 to preform a sample measurement, because the ratio of a two-channel detector response is expected to be directly proportional to the property of interest that the MOE is designed to measure.

## 3. Experiments Setup and Training Spectral Data Collection

### 3.1. Instrument and Experiment Setup

**Figure 3:**
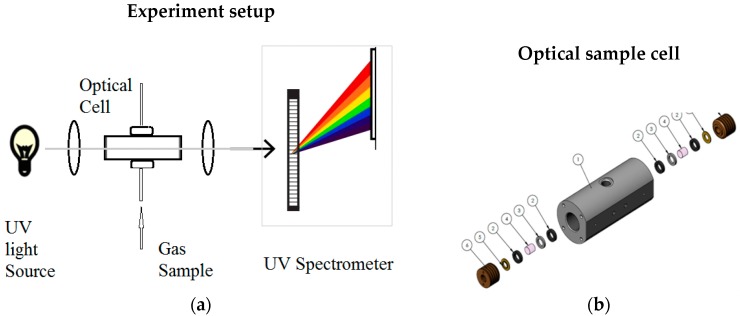
(**a**) Schematic setup of UV spectra collection experiment; (**b**) mechanical drawing of optical cell.

The experiment setup for UV spectral data collection is illustrated in Figure 3a. The setup had been previously described with slight modifications [20,27] and consisted of a UV light source (deuterium lamp (D2), low pressure 5 Torrs (0.1 psi), Hamamatsu Inc.) , two convex lenses (Edmund Optics, Barrington, NJ, USA), a high-pressure/high-temperature (HPHT) optical cell with CaF_2_ window (thickness of 1 cm) on each sides (custom built by Halliburton company, Houston, TX, USA) , an optical fiber (Ocean Optics), and a UV spectrometer (Ocean Optics HR200, Dunedin, FL, USA). The deuterium lamp was used as a broadband UV source. The radiation of the UV source was focused first by convex lens to collimate the light to pass through an HPHT optical cell (path length 10 cm) with two sapphire windows. The collimated beam is then focused by a second lens into an optical fiber, which is connected to a UV spectrometer. The spectrometer is controlled by a PC to record spectral data of the gas sample in the optical cell. The spectral range of the spectrometer is 165–1100 nm, with a spectral resolution of 1 nm. Figure 1b illustrates the design of HPHT optical cell with two sapphire windows. The HPHT optical cell can be pressurized up to 15,000 psi. For high-temperature experiments, the optical cell can be placed into a temperature-controlled oven to heat up to 300 °F.

### 3.2. Spectral Data Collection

**Figure 4:**
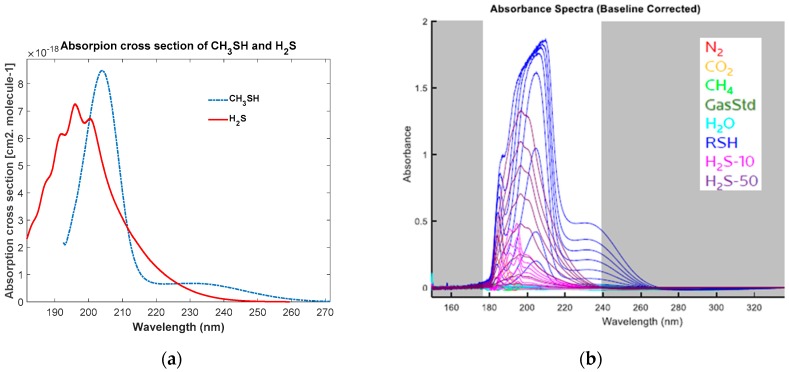
(**a**) Reference UV absorption cross sections of methyl mercapan CH_3_SH and hydrogen sulfide H_2_S at room temperature, the cross sections data were retrieved from the MPI-Mainz UV–vis Spectra Atlas [19,21]; (**b**) UV absorbance spectra of H_2_S (10 ppm and 50 ppm), RSH (CH_4_SH, 50 ppm), CO_2_, H_2_O, N_2_, and hydrocarbon gases mixture (GasStd), note that H_2_S (10 ppm and 50 ppm), CH_4_SH (50 ppm) were measured at multiple pressure at various pressures conditions from 96.5 kPa (14 psi) to 6894.7 kPa (1000 psi), resulting in multiple spectra in the figure.

One of challenges of designing an MOC optical sensor for gas-phase H_2_S measurements is the spectral interference from other compounds in the gas mixture. In the oil and gas exploration and production environment, for downhole or surface gas measurements, potential interfering compounds are hydrocarbon gases (methane, ethane, propane, butane, and pentane); CO_2_; H_2_O vapor; volatile aromatics which include benzene, toluene, ethylbenzene, and xylene (BTEX); and mercaptans. The absorption cross sections of H_2_S and methyl mercaptan (CH_3_SH) are retrieved from the MPI-Mainz UV–vis spectral Atlas database [19,21] and shown in Figure 4a. Using the experiment setup, UV spectra of hydrocarbon mixtures, CO_2_, H_2_O, BTEX, and methyl mercaptan (CH_4_SH) were also collected in the lab. The UV spectra of these compounds described above, with exception to the BTEX, are shown in Figure 4b. In the spectral range of 185 to 240 nm, where H_2_S has strong absorption, only methyl mercaptan and CO_2_ have absorbance peaks that overlay with the absorbance peak of H_2_S. Therefore, the spectra of the mixtures of H_2_S, CO_2_, and CH_4_SH were collected and used as training spectra for MOE design. Once the spectral data are available, a chemometric analysis is conducted to evaluate the performance of quantitative linear regression models, such as PLS (partial least squares) or PCR (principal component regression). If the linear regression model is deemed to meet measurement accuracy/precision requirements, MOE design can be carried out to design an optimal MOE to achieve similar performance as the linear calibration model.

#### 3.2.1. Low-Pressure and Room-Temperature UV Spectral Data Collection

**Figure 5:**
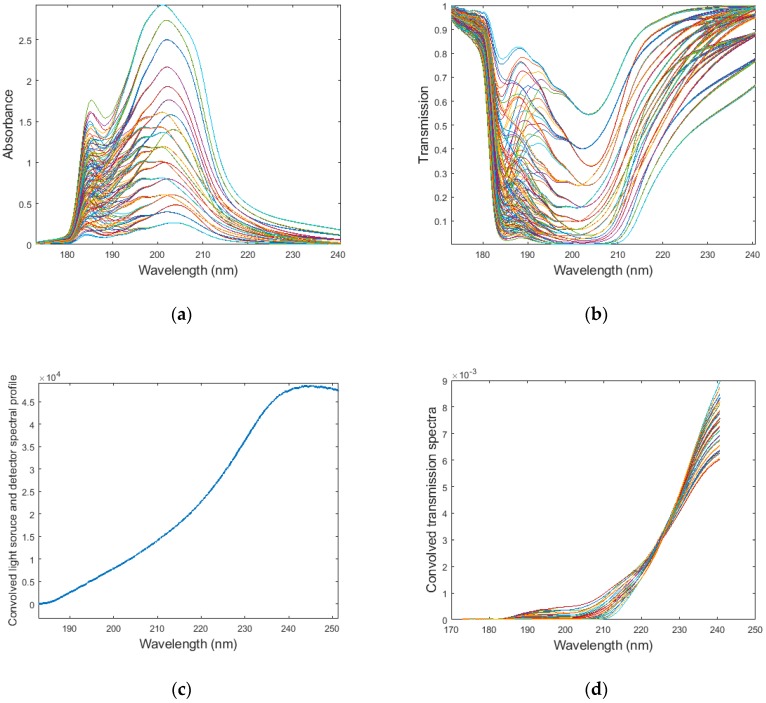
(**a**) UV absorbance spectra of H_2_S and CH_4_SH mixtures (H_2_S concentration varies from 7 nmol/mL to 141 nmol/mL); (**b**) UV transmission spectra of H_2_S and CH_4_SH mixtures; (**c**) convolved spectral profile of light source and detector; (**d**) system spectral profile convolved transmission spectra of samples.

To design an MOE for the MOC sensor, spectral data of various samples (H_2_S, CO_2_, and CH_4_HS mixtures with varying concentrations of each compound in N_2_ buffer gas) need to be collected as a spectral data training set. For a low-pressure and room-temperature (25 °C) spectra experiment, various mixture samples of H_2_S, CO_2_, and CH_4_SH in N_2_ buffer gas were measured under eight pressure set points [from 96.5 kPa (14 psi) to 6894.7 kPa (1000 psi)], resulting in a total number of 132 UV spectra after removing some outlier spectra. After each experiment on the H_2_S mixture sample, the sample cell was flushed by N_2_ gas for 10 mins, and the background spectrum was recorded as a reference spectrum. The transmission and absorbance spectra of the samples are shown in Figure 5a,b, respectively.

The transmission spectra of H_2_S-containing mixtures were convolved with a D2 light source emission spectral profile. The convolved spectra are shown in Figure 5d.

#### 3.2.2. High-Pressure/High-Temperature UV Spectral Data Collection

HPHT experiments were also conducted in the same experiment setup as shown in Figure 3a. The only difference between room-temperature and high-temperatures experiments is that the optical cell is placed into an oven with programmable temperature control. Before running the H_2_S samples, N_2_ gas was pumped to the cell to reach high-pressure setpoints (20.68, 41.37, 60.05, 82.73, and 103.42 MPa; or 3, 6, 9, 12, and 15 kpsi), and UV background spectra were recorded at five temperatures (65.6, 93.3, 121.1, 148.9, and 176.7 °C; or 150, 200, 250, 300, and 350 °F). These spectral data will be used as reference spectra. The same procedure was used to sequentially measure UV spectral profiles of H_2_S samples of 10 ppm and 50 ppm after N_2_ reference measurements. After finishing the H_2_S samples, the N_2_ sample experiment was repeated to record the N_2_ reference spectra at the same set of T & P setpoints. The H_2_S transmission spectra can be calculated through dividing the spectral profiles of H_2_S samples by the N_2_ reference spectra at corresponding temperature and pressure setpoints.

**Figure 6:**
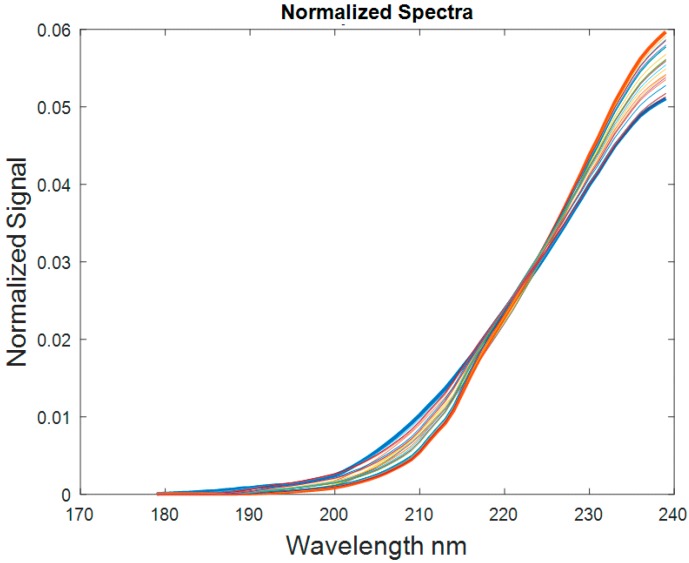
Normalized spectra of 10 ppm H_2_S sample at various high temperatures and high pressures (pressures: 20.68, 41.37, 60.05, 82.73, and 103.42 MPa; temperatures: 65.6, 93.3, 121.1, 148.9, and 176.7 °C).

The normalized spectra of samples with 10 ppm H_2_S are shown in Figure 6 below. The spectra region of interest is from 175 to 240 nm. Because one spectrum was acquired at every setpoint (temperature and pressure combination), 25 spectra (5T by 5P) were acquired for each sample.

## 4. Design and Fabrication of Multivariate Optical Element

**Figure 7:**
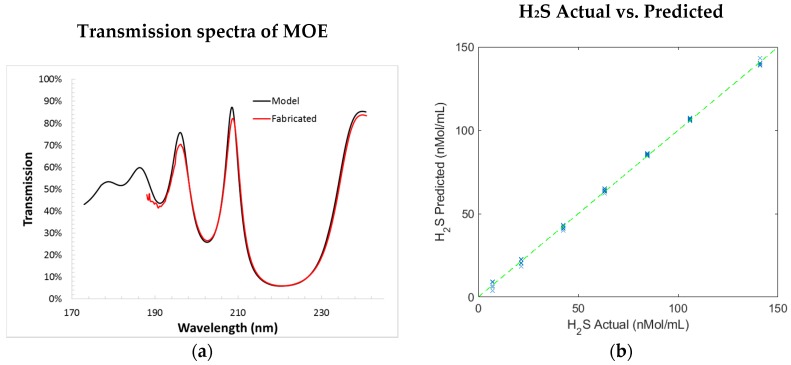
(**a**) Comparison of the simulated spectrum of the designed MOE and the measured spectrum of the fabricated MOE, two spectra match very well; (**b**) the expected calibration performance of the fabricated MOE.

Based on the UV spectral data collected from both room-temperature/low pressure and high-temperature/high-pressure experiments, an H_2_S-sensing UV MOE design search was performed using the optimization algorithm outlined in Equation (5). The optimal MOE film stack includes a fused silica as a filter substrate (1 mm thickness) and 22 alternating layers of Al_2_O_3_ and SiO_2_ (thickness of each layer varies from 10 nm to 200 nm, total film thickness is 1527 nm). The MOE was fabricated using an ion-assisted E-beam deposition method as previously described [39]. Figure 7a shows the transmission spectrum of the fabricated MOE and the simulated spectrum of the designed MOE. As demonstrated in Figure 7a, these two spectra matches very well, which indicates a successful fabrication. The calibration performance of MOE, based on projecting the fabricated MOE’s transmission spectrum to the samples’ training spectra, is shown Figure 7b. The standard error of calibration (SEC) calculated using the fabricated MOE is 3.3 nanomole per milliliter (nmol/mL, or micromolar); the relative accuracy (relative SEC) is ~2.2%; and the detection sensitivity (relative signal change) is ~10% for an H_2_S concentration ranging from 0 to 150 nmol/mL.

## 5. Multivariate Optical Computing Sensor Test 

### 5.1. Prototype MOC System Testing Setup 

**Figure 8:**
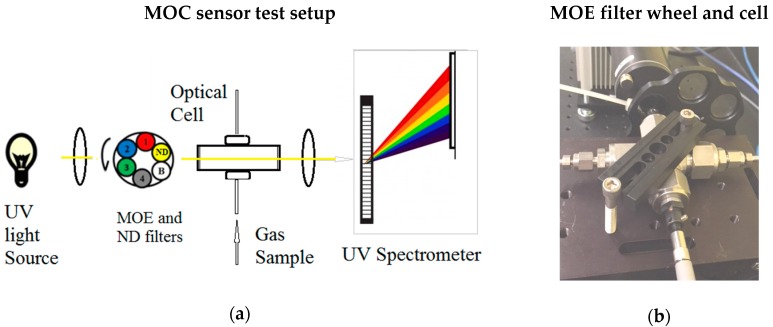
(**a**) Schematic setup of MOC prototype system for H_2_S test, channel number 1 to 4 are replicates of MOE, channel B is blocker channel to block the light, channel ND includes a 25% neutral density filter; (**b**) the picture of experiment setup.

The fabricated H_2_S MOE filters and ND filters were assembled to a rotating wheel, which was inserted into the optical path between the first collimating lens and optical sample cell, as shown in Figure 8. By rotating the wheel, a light beam passes the MOE filter and ND filter sequentially, and two signals from the detector were generated. The ratio of two signals (one through MOE, another through ND filter) was used to estimate the concentration of H_2_S. The integrated signal of the HR2000 spectrometer was used as opposed to a simple photodiode, due to delayed arrival of a short pass filter (250 nm). Without the short pass filter, a simple photodiode cannot be used due to long wavelength light contamination, HR2000 spectrometer’s grating and detector array were used as alternative detector because the grating allow us control desired spectral region to be integrated. For future commercial MOC sensor development, the short pass filter and simple photodiode should be used to design a simple and more robust MOC sensor.

### 5.2. MOC System Testing Results

Three gas samples were used to test the prototype instrument under eight pressure setpoints (from 14 to 1000 psi). At each pressure setpoint, two sets of spectral intensity data from the UV spectrometer were sequentially recorded. The MOE filter was first placed in the optical path, and a set of 10 spectral intensity data was recorded. The filter wheel was then rotated to place the ND filter in the optical path, and another set of 10 spectral intensity data was recorded. The spectral intensity data were then integrated from 180 to 240 nm to obtain the integrated intensity. The UV spectrometer is essentially used as a diode detector to measure the light intensity after light passes through the filter (either MOE or ND filter) and sample. The ratio of the integrated detector signal from the MOE channel to the integrated detector signal from the ND channel was calculated for each pressure setpoint; the ratio was defined as A/B ratio. The optical cell was flushed with N_2_ gas for 30 minutes between sample runs.

#### 5.2.1. H_2_S Gas Sample Test under Different Pressures

**Figure 9:**
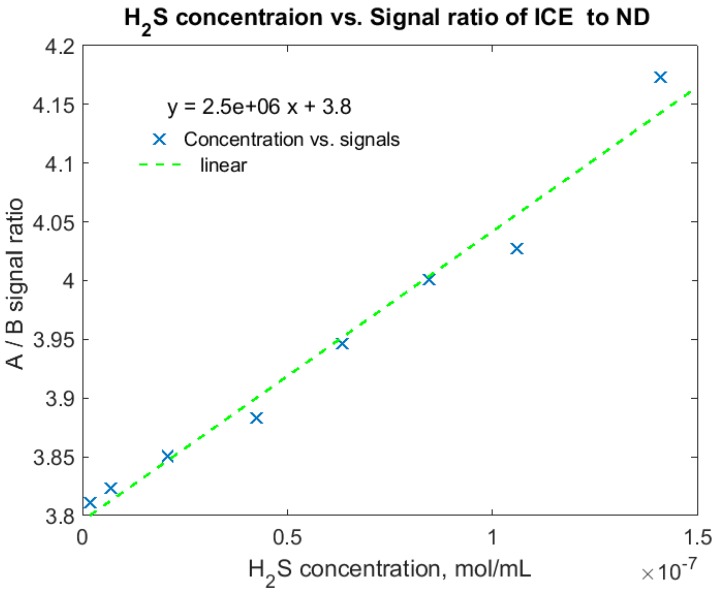
Calibration curve that correlates H_2_S concentrations to A/B signal ratios, using 50 ppm H_2_S under eight pressure setpoints.

A gas sample with 50 ppm H_2_S in N_2_ buffer gas was tested first. The concentration of H_2_S increased proportionally with pressure within the pressure range of 14 to 1000 psi. Figure 9 shows the A/B ratio vs. H_2_S concentrations in mol/mL, in which the A/B ratio increases linearly with the concentration of H_2_S in the sample cell (coefficient of determination R^2^ = 0.97, SEC = 4.5 nmol/mL). The A/B ratio increases from 3.8 to 4.2 for the H_2_S concentration range, yielding a relative signal change of 10%, which matches well with the expected sensitivity of MOE design. A calibration curve was developed based one the relationship of the A/B ratios and the H_2_S concentrations. The calibration curve was then used to predict the concentration of H_2_S in the following two H_2_S mixture measurements.

#### 5.2.2. H_2_S and CH_3_SH Gas Mixture Samples Test under Different Pressures

**Figure 10:**
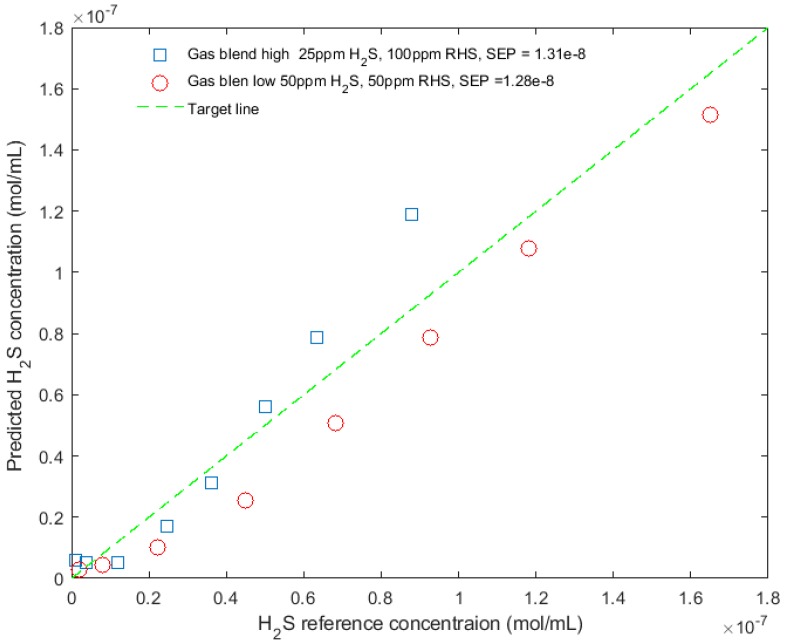
Prediction vs. reference of H_2_S concentration of two mixture samples (H_2_S and CH_3_SH mixture) at eight pressure setpoints using the MOC prototype sensor.

In addition to the pure H_2_S in the N_2_ buffer gas experiment, another two samples containing H_2_S and CH_3_SH mixtures in N_2_ buffer gas were used to test the prototype sensor. One sample contained 25 ppm H_2_S and 100 ppm CH_3_SH, while the other sample contained 50 ppm H_2_S and 50 ppm CH_3_SH. The same experiment protocol and data processing method, as described in the last section, was used to acquire and process the data generated from the gas mixture experiments. The calibration curve developed in the last experiment was used to predict the H_2_S concentration. Figure 10 shows the reference versus the predicted H_2_S concentration for these samples under eight pressure setpoints. The standard error of prediction (SEP) for the sample with 25 ppm H_2_S and 100 ppm CH_3_SH under various pressures is 13.1 nmol/mL, and the SEP for the other sample is 12.8 nmol/mL. The average SEP for all samples under various pressures is 12.9 nmol/mL, resulting in a relative prediction accuracy (relative SEP) of 7.9%. The sensor performances at each stage of sensor development are summarized in Table 1.

## 6. Conclusions

This paper described a sensor design and analytical method to quantitatively measure the H_2_S concentration in H_2_S-containing gas mixtures at various pressures by using an MOC-based sensor. To design the sensor, a sample cell and spectrometer system was set up to collect UV spectra of stationary, non-flowing gas mixtures at various temperatures and pressures. The spectra were later used as training spectra to design an MOE filter to be fabricated and assembled into an MOC prototype sensor. To test the analytical method and MOC sensor performance, three samples with known H_2_S concertation were used. Each sample was pumped to various pressures (from 14 to 1000 psi), and sensor responses were recorded and analyzed. The first sample containing 50 ppm H_2_S in N_2_ gas was tested using the MOC sensor, and a linear relation was discovered between processed sensor responses and H_2_S concentrations. The accuracy and sensitivity of the measurements match well with the sensor’s design performance. In addition, two gas mixture samples, which contain both target compound H_2_S and interference compound CH_3_SH at different ratios, were used to test the MOC sensor. The performance of the MOC sensor demonstrated that it can achieve an analytical accuracy better than 10% for H_2_S measurement at various pressures despite substantial spectral interference from CH_3_SH, because the MOE was designed to work against the spectral interference and to tailor the signals to be proportional to H_2_S concentrations.

A number of factors can contribute to the MOC sensor prediction error. First, the reference concentrations of the H_2_S samples were calculated based on the ideal gas assumption, as higher pressure increases the H_2_S concentration proportionally. This ideal gas assumption might result in an inaccurate reference H_2_S concentration calculation. An equation-of-state (EoS) model should be used for a more accurate calculation of reference H_2_S concentration. Second, the uncertainty of pressure control of the experiment is estimated to be about 5%, which can contribute to the calculated SEC and SEP.

## Figures and Tables

**Table 1:** Summary of MOE performance.

Performance	Design MOE	Fabricated MOE	Sample 1 (50 ppm H_2_S)	Sample 2 (25 ppm H_2_S and 100 ppm CH_3_SH Mixture)	Sample 3 (50 ppm H_2_S and 50 ppm CH_3_SH Mixture)
Simulation or MOC test	Simulation	Simulation	Calibration/Test	Test	Test
SEC (nmol/mL)	2.8	3.3	4.5	-	-
Relative SEC (%)	1.8	2.2	3.0	-	-
SEP (nmol/mL)	-	-	-	13.1	12.8
Relative SEP (%)	-	-	-	8.1	8.0
Relative sensitivity ^1^ (%)	10.5	10	10	10.5	9.8

^1^ Calculated based on A/B ratio signal difference divided by signal mean for 0 to 150 nmol/mL H_2_S.
